# Using Genomics to Track Global Antimicrobial Resistance

**DOI:** 10.3389/fpubh.2019.00242

**Published:** 2019-09-04

**Authors:** Rene S. Hendriksen, Valeria Bortolaia, Heather Tate, Gregory H. Tyson, Frank M. Aarestrup, Patrick F. McDermott

**Affiliations:** ^1^European Union Reference Laboratory for Antimicrobial Resistance, World Health Organisation, Collaborating Center for Antimicrobial Resistance and Genomics in Food borne Pathogens, FAO Reference Laboratory for Antimicrobial Resistance, National Food Institute, Technical University of Denmark, Lyngby, Denmark; ^2^Center for Veterinary Medicine, Office of Research, United States Food and Drug Administration, Laurel, MD, United States

**Keywords:** global, antimicrobial resistance, surveillance, genomic, bioinformatics tools, microbiology

## Abstract

The recent advancements in rapid and affordable DNA sequencing technologies have revolutionized diagnostic microbiology and microbial surveillance. The availability of bioinformatics tools and online accessible databases has been a prerequisite for this. We conducted a scientific literature review and here we present a description of examples of available tools and databases for antimicrobial resistance (AMR) detection and provide future perspectives and recommendations. At least 47 freely accessible bioinformatics resources for detection of AMR determinants in DNA or amino acid sequence data have been developed to date. These include, among others but not limited to, ARG-ANNOT, CARD, SRST2, MEGARes, Genefinder, ARIBA, KmerResistance, AMRFinder, and ResFinder. Bioinformatics resources differ for several parameters including type of accepted input data, presence/absence of software for search within a database of AMR determinants that can be specific to a tool or cloned from other resources, and for the search approach employed, which can be based on mapping or on alignment. As a consequence, each tool has strengths and limitations in sensitivity and specificity of detection of AMR determinants and in application, which for some of the tools have been highlighted in benchmarking exercises and scientific articles. The identified tools are either available at public genome data centers, from GitHub or can be run locally. NCBI and European Nucleotide Archive (ENA) provide possibilities for online submission of both sequencing and accompanying phenotypic antimicrobial susceptibility data, allowing for other researchers to further analyze data, and develop and test new tools. The advancement in whole genome sequencing and the application of online tools for real-time detection of AMR determinants are essential to identify control and prevention strategies to combat the increasing threat of AMR. Accessible tools and DNA sequence data are expanding, which will allow establishing global pathogen surveillance and AMR tracking based on genomics. There is however, a need for standardization of pipelines and databases as well as phenotypic predictions based on the data.

## Introduction

The science of infectious disease, along with other medical and biological specialties, is undergoing rapid change brought on by the advent of affordable whole genomic sequencing (WGS) technologies (1–3). These technologies are rapidly gaining acceptance as routine methods, and in the process, are transforming laboratory procedures.

The amount of bacterial genomic data being generated is immense. As of this writing, for example, over 190,000 *Salmonella* genomes alone are in the public domain with hundreds being added weekly. A complete genomic DNA sequence represents the highest practicable level of structural detail on the individuating traits of an organism or population. As such, it can be used to provide more reliable microbial identification, definitive phylogenetic relationships, and a comprehensive catalog of traits relevant for epidemiological investigations. This is having a major impact on outbreak investigations and the diagnosis and treatment of infectious diseases, as well as the practice of microbiology and epidemiology (4). Furthermore, DNA sequences are a universal dataset from which, theoretically, any biological feature can be inferred. In clinical applications, this includes the ability to detect antimicrobial resistance (AMR), and to track the evolution and spread of AMR bacteria in a hospital or the community.

AMR is a global health problem that contributes to tens of thousands of deaths per year [Chaired by Jim O'Neill, (5)]. Historically, AMR has been detected as a measurement of the growth inhibitory effects of a chemotherapeutic agent on a bacterial population cultured under specific laboratory conditions. Despite some ancillary enhancements, clinical laboratories to this day rely mainly on diffusion and dilution methods to guide clinical therapy and to monitor AMR over time. Accumulating data show that AMR can be accurately predicted from the genomic sequence for many bacteria. The sequence-based approach to AMR detection requires robust bioinformatics tools to analyze and visualize the genomic structure of the microbial “resistome,” defined by AMR genes and their precursors (6). This review summarizes the state of the science in using single isolate WGS to track global AMR.

## The Advantages of Whole Genome Sequencing

A major advancement enabling resistome surveillance is the demonstrated power to predict AMR from genomic data alone. Several studies including those focused on foodborne pathogens and *Enterobacteriaceae* have shown a high concordance (>96%) between the presence of known AMR genes or mutations and Minimum Inhibitory Concentration (MIC) of several antimicrobials at or above the epidemiological cut-off value or clinical breakpoint for resistance. High sensitivity of >87%, defined by the ability to correctly identify AMR determinants associated with an antimicrobial resistance phenotype (true positive rate) and high specificity of >98%, defined by the ability to correctly identify the absence of AMR determinants in an antimicrobial susceptible phenotype (true negative rate), have been observed depending on the bacterial species analyzed (Table 1) (7–18). Furthermore, a growing body of data shows that it is possible to predict AMR, and perhaps the MIC of an antimicrobial, applying machine or deep learning to genome sequence data (19–21). The comparison between phenotype and genotype as well as the application of machine or deep learning are however still in their infancy and additional data on bacterial species beyond the foodborne pathogen domain are needed.

**Table 1:** Concordance between phenotypic susceptibility testing and WGS based predicted antimicrobial resistance.

	**Pathogen**	**No. of pathogens**	**AST method**	**No. of antimicrobials**	**Bioinformatic tool**	**Sequencing data**	**Concordance**	**Sensitivity**	**Specificity**	**Comment**	**References**
2013	*S. Typhimurium*	49	MIC	17	ResFinder	Assembled, Velvet	99.74%			Disagreement: 7 isolates including 6 *E. coli* resistent to Spec	(7)
	*E. coli*	48									
	*E. faecalis*	50		14							
	*E. faecium*	50									
2013	*E. coli* (ESBL)	74	DD	7	BLASTn, selected panel	Assembled, Velvet		96%	97%	VM rate: 1.2%/M rate: 2.1%	(8)
	*K. pneumonia* (ESBL)	69									
2014	*S. aureus*	501	DD/MIC (Vitek)	12	BLASTn, selected panel	Assembled, Velvet		97%	99%	VM rate: 0.5%/M rate: 0.7%	(9)
2016	*C. jejuni*	32	MIC	9	BLASTx	Assembled, CLC-bio	99.2%			Lower concordance to	(10)
	*C. coli*	82								Gen, Azi, Clin, Tel	
2016	*S. enterica*	104	MIC	14	ResFinder/ ARG-ANNOT/ CARD/BLAST	Assembled, CLC-bio	99.0%	99.2%	99.3%	Lower concordance to	(11)
		536						97.6%	98.0%	aminoglycosides/β-lactams	
2017	*E. coli*	31	MIC	4	Custom DB based on ARDB/CARD/β-lactamase allelles			87%	98%	Neg. predictive value: 97%	(12)
	*K. pneumonia*	24								Pos. Predictive value: 91%	
	*P. aeruginosa*	22									
	*E. cloacae*	13									
2017	*S. enterica*	50	MIC	4	ResFinder/ PointFinder	Assembled, SPAdes	98.4%			Disagreement: 2/2 C.jejuni to FQ/ERY	(13)
	*E. coli*	50		6							
	*C. jejuni*	50		4						5 *E. coli* to COL (pmrB)	
2018	*E. faecalis*	97	MIC	11	ResFinder/NCBI Pathogen DB/BLAST	Assembled, CLC-bio	96.5%				(14)
	*E. faecium*	100									
2018	*S. aureus*	501	DD/MIC	12	GeneFinder/ Mykrobe/ Typewriter	FASTQ/assembled, BLAST	98.3%			Disagreements: 0.7% predicted resistant	(15)
		491									
		397	MIC							0.6% predicted susceptible	
2018	*M. tuberculosis*	10,209	MGIT 960	4	Cortex	Assembled	89.5%			97.1%/99.0% predicted R/S	(16)
				4						97.5%/98.8% predicted R/S	
				4						94.6%/93.6% predicted R/S	
				4						91.3%/96.8% predicted R/S	
2019	*H. pylori*	140	MIC (E-test)	5	ARIBA	FASTQ	99%			Phenotype issues to metronidazole	(17)

*1) ESBL: Extended Spectrum Beta-Lactamase, 2) MIC: Minimum Inhibitory Concentration, 3) DD: Disk diffusion, 4) VM: Very Major, 5) M: Major, 6) R/S: Resistant/Susceptible, 7) SPEC: Spectinomycin, 8) GEN: Gentamicin, 9) AZI: Azithromycin, 10) CLIN: Clindamycin, 11) TEL: Telithromycin, 12) FQ: Fluoroquinolone, 13) ERY: Erythromycin, 14) COL: colistin*.

The most obvious advantage of WGS for microbial typing and AMR surveillance is the unprecedented level of detail in one assay that can be used to describe current trends and distinguish emerging tendencies (22). AMR bacteria can be typed and traced by specific allele profiles, rather than just according to phenotypic patterns by drug class. This is exemplified by a study of emerging aminoglycoside-resistant *Campylobacter* in the USA, where WGS revealed that the rising trend was driven by nine different resistance alleles, six of which had never been detected in *Campylobacter* previously and would not have been found easily using PCR (10). Similarly, in one of the first large-scale applications of WGS to investigate a drug-resistant foodborne outbreak in the US in 2011, inconsistent resistance patterns among indistinguishable PFGE types of *Salmonella* serovar Heidelberg were revealed by sequence analysis to be a polymicrobic contamination event, involving various combinations of plasmids and strain types (23).

DNA sequence-based surveillance makes it possible also to define multidrug-resistance (MDR) with much greater precision compared to phenotypic tests (22). It has long been a common practice to define MDR as resistance to compounds from three or more drug classes (24), a definition with limited practical value. Bioinformatic analysis can reveal the co-carriage of specific genes underlying different MDR patterns, allelic trends over time, their genetic context including the potential for horizontal transfer, and their distribution by source. In addition, the presence of co-resistances not assayed on standard drug panels is revealed, such as disinfectant and heavy metal resistance. This level of “deep surveillance” can uncover other potential drivers of AMR persistence and evolution, and the opportunity for a more refined microbial risk analysis based on the association of resistance traits with specific sources.

## Online Resources for *in silico* Antimicrobial Resistance Detection

**Figure 1:**
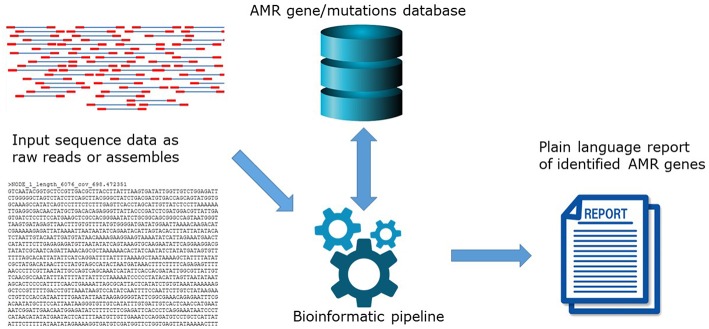
The principle of *in silico* AMR determinant detection using a search algorithm to query input DNA.

The high level of agreement between phenotype and genotype coincides with the development of new and updated versions of bioinformatics tools to predict AMR, and the maturation of well-curated AMR gene databases. In principle, *in silico* AMR detection is performed by using a search algorithm to query input DNA or amino acid sequence data for the presence of a pre-determined set of AMR determinants contained in AMR reference databases (Figure 1). This can be performed using proprietary systems offered by commercial companies or open-access systems requiring different levels of user expertise. Open-access systems are available at public genome data centers such as the Center for Genomic Epidemiology (CGE) http://www.genomicepidemiology.org/ online or downloadable for local install from github (https://github.com/), bitbucket (https://bitbucket.org/account/user/genomicepidemiology/projects/DB) and similar.

The various bioinformatics software can process sequence data either as reads or as assemblies (25). Generally, available resources do not include quality control of input sequence data thus it is the users' responsibility to ensure the quality of submitted sequences or assemblies. When using assembly-based methods, differences among assemblers may compromise comparability of the outcome (15, 26). Following assembly, the most common approaches to compare the input data with the AMR reference databases rely on BLAST and Hidden Markov Model searches, among others. BLAST-based tools can give different outputs based on default settings for gene length and percentage of similarity. This can negatively affect specificity if the settings are too low or too high. Moreover, assembly-based methods are computationally demanding. Despite these caveats, assembly-based methods may have an added value in an AMR surveillance context as they allow analysis of the genetic context of the AMR genes such as their presence on mobilizable potential. Read-based methods may use different tools to align reads to AMR databases, including Bowtie2, BWA, and KMA (25). Recently, the KMA (k-mer alignment) has been develop to map raw reads directly against redundant AMR databases (27). The KMA tool was developed specifically for rapid and accurate bacterial genome analyses in contrast to other mapping methods such as BWA that were developed for large reference genome, such as the human genome and subsequently applied empirically to microbiology (27). KMA uses k-mer seeding to speed-up mapping and the Needleman-Wunsch algorithm to accurately align extensions from k-mer seeds. Multi-mapping reads are resolved using a novel sorting scheme (ConClave scheme) to ensure an accurate selection of templates (27). Read-based methods allow identification of AMR genes present in low abundance which might be overlooked where assemblies are incomplete (25).

Independent of the bioinformatics approach chosen, the performance of *in silico* AMR prediction is critically dependent on the availability of accurate AMR databases. AMR reference databases can be subdivided into solutions specialized for detection of resistance to specific antimicrobials and/or in specific bacterial species or in solutions allowing detection of virtually any possible AMR determinant in any DNA/amino acid sequence. Besides their focus area, AMR reference databases have important differences which users need to acknowledge for choosing the optimal fit-for-purpose database. First, AMR reference databases differ for criteria of inclusion of entries. For example, entries in CARD must have been published in scientific literature. In ResFinder, publication is not a strict requirement. Genes must have a GenBank number and expert review of the GenBank entries. Also, the types of entries differ across databases, with most databases including AMR genes and only a few databases including mutations of chromosomal genes mediating AMR. Finally, the available AMR databases differ regarding the format of the entries (fasta, json, etc.), the possibility of download, and the availability and frequency of curation (Table 2).

**Table 2:** Open-access resources for *in silico* antimicrobial resistance detection in bacteria.

**Name**	**Target**	**Software**		**Database**		**Input sequence**		**Link**	**Year of development**	**Curation (last update)**	**References**
		**Type**	**Downloadable*^a^***	**Source**	**Downloadable**	**Type**	**Format**				
ABRES Finder	General AMR	Profile HMM	No	Own	No	Amino acid	FASTA	http://scbt.sastra.edu/ABRES/index.php	2017	Not specified	Unpublished
ABRICATE	General AMR	BLAST	Yes	ResFinder, CARD, ARG-ANNOT, NCBI AMRFinder, EcOH, PlasmidFinder, Ecoli_VF and VFDB	Yes	Nucleotide	FASTA	https://github.com/tseemann/abricate	2016	2019	Unpublished
ARDB	General AMR	BLAST	Yes	Own	Yes	Nucleotide	FASTA	https://ardb.cbcb.umd.edu/	2009	2009	(28)
ARG-ANNOT	General AMR	–	–	Own	Yes	–	–	Discontinued	2014	2018	(29)
ARIBA	General AMR (single isolate sequences)	Minimap, Bowtie2	Yes	Derived from ARG-ANNOT, CARD, PlasmidFinder, ResFinder, VFDB^b^; customizable	No	Nucleotide	FASTQ	https://github.com/sanger-pathogens/ariba	2017	2019	(30)
CARD	General AMR	BLAST, RGI	Yes	Own	Yes	Nucleotide, amino acid	FASTA	https://card.mcmaster.ca/home	2013	2019	(31)
IRIDA plugin AMR detection	General AMR	RGI, staramr	Yes	CARD, PointFinder, PlasmidFinder and ResFinder	Yes	Nucleotide	FASTQ	https://github.com/phac-nml/irida-plugin-amr-detection	2019	2019	Unpublished
Kmer resistance	General AMR	KMA	Yes	ResFinder	Yes	Nucleotide	FASTA, FASTQ	https://cge.cbs.dtu.dk/services/KmerResistance-2.2/	2016	2019	(26)
MEGARes (AMRplusplus)	General AMR	BWA	Yes	Derived from ARG-ANNOT, CARD, NCBI Lahey Clinic beta-lactamase archive, ResFinder^b^	Yes	Nucleotide	FASTQ	https://megares.meglab.org/	2016	2016	(32)
NCBI AMRFinder	General AMR	BLAST, HMMER	Yes	Own	Yes	Nucleotide, amino acid	FASTA, GFF	https://www.ncbi.nlm.nih.gov/pathogens/antimicrobial-resistance/AMRFinder/	2017	2019	(33)
Noradab	General AMR	BLAST	No	Derived from ARDB and CARD^b^	Yes	Nucleotide, amino acid	FASTA	http://noradab.bi.up.ac.za/	2018	Not specified	(34)
Patric	General AMR	BLAST	Yes	Own	Yes	Nucleotide, amino acid	FASTA	https://www.patricbrc.org/	2004	2019	(35)
ResFinder	General AMR	BLAST, KMA	Yes	Own	Yes	Nucleotide	FASTA, FASTQ	https://cge.cbs.dtu.dk/services/ResFinder/	2012	2019	(36)
SRST2	General AMR	BOWTIE2	Yes	Derived from ARG-ANNOT^b^	Yes	Nucleotide	FASTA, FASTQ and any other format readable by BOWTIE2	https://github.com/katholt/srst2	2014	2019	(37)
SSTAR	General AMR	BLAST	Yes	Derived from ARG-ANNOT and Resfinder^b^	Yes	Nucleotide	FASTA	https://github.com/tomdeman-bio/Sequence-Search-Tool-for-Antimicrobial-Resistance-SSTAR-	2015	2018	(38)
INTEGRALL	AMR genes and associated integrons	BLAST	No	Own	Yes	Nucleotide	FASTA	http://integrall.bio.ua.pt/?	2008	2019	(39)
MvirDB	AMR genes, protein toxins and virulence factors for bio-defense applications	BLAST	No	Derived from Tox-Prot, SCORPION, the PRINTS virulence factors, VFDB, TVFac, Islander, ARGO and a subset of VIDA^b^	Yes	Nucleotide, amino acid	FASTA	Discontinued (http://mvirdb.llnl.gov/)	2007	Not specified	(40)
BacMet	Biocide and metal resistance	BLAST	No	Own	Yes	Nucleotide, amino acid	FASTA	http://bacmet.biomedicine.gu.se/	2013	2018	(41)
ResCap	Antibiotic, heavy metal and biocide resistance	BLAST, Bowtie2	Yes	Derived from ARG-ANNOT, CARD, RED-DB, ResFinder, Bacmet^b^	Yes	Nucleotide	FASTA, FASTQ	https://github.com/valflanza/ResCap	2017	2017	(42)
ARGO	Beta-lactam and vancomycin resistance	–	–	Own	–	–	–	Discontinued (http://bioinformatics.org/argo/beta/antibioticresistance.php)	2005	–	(43)
RED-DB	Beta-lactam, glycopeptide, aminoglycoside, tetracycline, sulphonamide, macrolide, lincosamide, streptogramin b, oxazolidinone and quinolone resistance	BLAST	No	Own	Yes	Nucleotide, amino acid	FASTA	http://www.fibim.unisi.it/REDDB/	2007-2013	Not specified	Unpublished
Tetracycline MLS nomenclature	Macrolide, lincosamide, streptogramin and tetracycline resistance	–	–	Own	Yes	–	–	https://faculty.washington.edu/marilynr/	Not specified	2019	Unpublished
β-lactamases Database	β-lactamases	–	–	Own	Yes	–	–	http://ifr48.timone.univ-mrs.fr/beta-lactamase/public/	Not specified	Not specified	Unpublished
BLAD	β-lactamases	–	–	Own	No	Nucleotide, amino acid	FASTA	http://www.blad.co.in/	2012	Not specified	Unpublished
BLDB	β-lactamases	BLAST	No	Own	Yes	Nucleotide, amino acid	FASTA	http://bldb.eu/	2017	2019	(44)
CBMAR	β-lactamases	BLAST	No	Own	Yes	Nucleotide, amino acid	FASTA	http://proteininformatics.org/mkumar/lactamasedb/	2014	2014	(45)
LacED	β-lactamases	BLAST	No	Own	Yes	Amino acid	FASTA	http://www.laced.uni-stuttgart.de/	2009	Not specified^c^	(46)
AMRtime	AMR genes in metagenomic data	DIAMOND	Yes	CARD	Yes	Nucleotide	FASTQ	https://github.com/beiko-lab/AMRtime	2017	2019	(47)
DeepARG	AMR genes in metagenomic data	BLAST, DIAMOND	Yes	Derived from RDB, CARD, UNIPROT^b^	Yes	Nucleotide, amino acid	FASTA, FASTQ	https://bench.cs.vt.edu/deeparg	2017	2019	(48)
GROOT	AMR genes in metagenomic data	LSH Forest indexing	Yes	Derived from ARG-ANNOT, CARD, Resfinder	Yes	Nucleotide	FASTQ	https://github.com/will-rowe/groot	2018	2019	(49)
SARG (ARGs-OAP; ARGpore)	AMR genes in metagenomic data	BLAST, HMMER, UBLAST	Yes	Derived from ARDB and CARD^b^	Yes	Nucleotide	any format is supported	https://smile.hku.hk/SARGs	2016	2019	(50)
SEAR	AMR genes in metagenomic data	BLAST, BWA-MEM	Yes	ARG-ANNOT	Yes	Nucleotide	FASTQ	Discontinued (https://github.com/will-rowe/SEAR)	2015	2018	(51)
ShortBRED	AMR genes in metagenomic data	BLAST, USEARCH	Yes	Derived from ARDB and CARD^b^	Yes	Amino acid	FASTA	http://huttenhower.sph.harvard.edu/shortbred	2015	2019	(52)
Mustard	AMR determinants in the human gut microbiota	BLAST	No	Derived from Resfinder, ARG-ANNOT, the Lahey Clinic (http://www.lahey.org/studies/), RED-DB (http://www.fibim.unisi.it/REDDB/), Marilyn Roberts' website for macrolides and tetracycline resistance (http://faculty.washington.edu/marilynr/) and different functional metagenomics studies^b^	Yes	Nucleotide, amino acid	FASTA	http://mgps.eu/Mustard/	2017	2017	(53)
FARMEDB	AMR genes discovered by functional metagenomics	BLAST	No	Own	Yes	Nucleotide, amino acid	FASTA	http://staff.washington.edu/jwallace/farme/index.html	2016	Not specified^c^	Unpublished
ResFams	AMR genes discovered by functional metagenomics	–	–	Derived from CARD, LacED, Lahey beta-lactamases (now at NCBI)^b^	Yes	–	–	http://www.dantaslab.org/resfams	2014	2018	(54)
ResFinderFG	AMR genes discovered by functional metagenomics	BLAST	Yes	Own	No	Nucleotide	FASTA, FASTQ	https://cge.cbs.dtu.dk/services/ResFinderFG-1.0/	2016	Not specified	Unpublished
Galileo AMR (MARA, RAC)	AMR genes in Gram-negative bacteria	BLAST (ATTACCA)	Yes	Own	Yes	Nucleotide	FASTA	https://galileoamr.arcbio.com/mara/	2017	Not specified3	(55)
LREfinder	Linezolid resistance in enterococci	KMA	Yes	Own	Yes	Nucleotide	FASTA, FASTQ	https://cge.cbs.dtu.dk/services/LRE-finder/	2019	2019	(56)
MUBII-TB-DB	AMR mutations in Mycobacterium tuberculosis	BLAST	No	Own	No	Nucleotide	FASTA	https://umr5558-bibiserv.univ-lyon1.fr/mubii/mubii-select.cgi	2013	Not specified	(57)
Mykrobe	AMR in Mycobacterium tuberculosis and Staphylococcus aureus	Own (based on de Bruijn graph)	Yes	Own	Yes	Nucleotide	FASTQ	http://www.mykrobe.com/	2015	2019	(58)
TBDReaM	AMR in Mycobacterium tuberculosis	–	–	Own	Yes	–	–	https://tbdreamdb.ki.se/Info/	2009	2014	(59)
PointFinder	Selected mutations in chromosomal genes of Escherichia coli, Salmonella sp., Campylobacter sp., Staphylococcus aureus, Enterococcus sp., Mycobacterium tuberculosis, Neisseria gonorrhoeae	BLAST, KMA	Yes	Own	Yes	Nucleotide	FASTA, FASTQ	https://cge.cbs.dtu.dk/services/ResFinder/	2017	2019	(13)
SCCmec Finder	SCCmec elements in Staphylococcus aureus	BLAST, KMA	Yes	Own	Yes	Nucleotide	FASTA, FASTQ	https://cge.cbs.dtu.dk/services/SCCmecFinder/	2016	2018	(60)
U-CARE	AMR in Escherichia coli	BLAST	No	Own	Yes	Amino acid	FASTA	http://www.e-bioinformatics.net/ucare/	2013	Not specified	(61)
ARGDIT	Toolkit for validation and integration of AMR gene database	–	Yes	–	–	Nucleotide, amino acid	FASTA	https://github.com/phglab/ARGDIT	2018	2019	(62)
ARG-miner	Robust and comprehensive curation of AMR gene databases	–	–	Derived from ARDB, ARG-ANNOT, CARD, DeepARG-DB, MEGARes, NDARO, ResFinder, SARG, UniProt^b^	Yes	–	–	https://bench.cs.vt.edu/argminer/#/home	2018	2019 (crowd-curation)	(48)

a *Yes, standalone version is available (usually in Bitbucket or in GitHub) either with or without a corresponding web version; no, only web version is available*.

b *Curation to avoid redundancies and remove selected sequences (see respective references for details)*.

c *Active, based on authors' knowledge; discontinued databases may still be available for download via WayBack Machine*.

At present, at least 47 online available resources for *in silico* AMR prediction are published in the scientific literature (13, 26, 28–63) (Table 2). They range from basic AMR reference databases that can be embedded in the user's own bioinformatics pipeline, to systems having a well-curated database with integrated search tools. These bioinformatics resources have interfaces of different complexity that require different skills in bioinformatics and microbiology for performing the sequence analyses and interpreting the results (Table 2). As the features of these systems differ widely, the outputs obtained by different tools may not be fully comparable. Moreover, employing the same tool for different input formats of the same data (e.g., raw reads vs. assembled sequences, trimmed vs. non-trimmed reads; assemblies obtained by different software, etc.) can produce different results (64). A reliable genomic approach to assaying AMR gene content requires accurate curated reference databases that should be synchronized and harmonized in a way to ensure comparable outputs worldwide. Once that is achieved, the bioinformatics method of monitoring will undeniably lead to a paradigm shift in the way that we conduct AMR surveillance and compare results internationally. Importantly, the currently available tools may detect new gene variants, but they are not presently equipped to detect new AMR genes. Identifying novel resistance elements from genomic data is being pursued using iterative kmer-based analytics and other machine learning schemes but these strategies still require well-characterized reference genomes with phenotypic data for training (11, 19–21).

## Benchmarking of Bioinformatics Tools to Detect Antimicrobial Resistance Determinants

Benchmarking exercises are important to assess the performance, and reliability of the available bioinformatics tools which have different complexity in design and function.

Designing and executing a benchmarking trial offers several challenges. At a recent meeting (October 2017) organized by the European Commission Joint Research Center, the challenges of designing a benchmarking strategy for assessing bioinformatics tools to detect AMR determinants was discussed (65). Here, several challenges were identified, and considerations discussed which included: (1) the origin of the dataset tested; (2) sustainable reference datasets; (3) quality of the test genomes; (4) what determinants to include in a dataset; (5) the, expected result; and (6) performance thresholds. The sequence dataset could either be real or artificially composed. In both cases, this will have implications for accurate benchmarking. A real dataset needs to be properly characterized and the true reference result defined. Furthermore, a real dataset may be biased in content for certain resistance determinants, such as mutations in the *ampC* promoter of *E. coli*, and thereby affect some bioinformatics tools more than others (26). In contrast, a simulated dataset needs to be accurate and correct but also contain a variety of different determinants or mechanisms. Ideally, a combination could be applied designing a desired benchmarking dataset to represent real-life scenarios aligned with the test objective (e.g., only focused on extended spectrum β-lactamases). The scope of bacterial species represented can also influence the results (65).

The quality and type of sequence data are also important factors. This also needs to mimic a real-life scenario where genomes will differ in error rates, read lengths, and read quality and may be raw reads or assemblies. The robustness of bioinformatics tools will differ in performance when dealing with low quality genomes and assemblies compared to optimal conditions (26, 65).

Prior to executing a benchmarking exercise, the reference AMR classes need to be determined as to whether all known or acquired determinants will be included, or only specific mechanisms such as certain enzymes, efflux pumps, mutations/single nucleotide polymorphisms (SNPs), upregulated or downregulated genes or porins. Ideally, the bioinformatics tools should enable the detection of all known determinants if used for surveillance or guiding clinical treatment unless the scope is different and agreed upon (65).

Since the main objective of a benchmarking exercise is to assess the ability of the bioinformatics tool to provide reliable analysis of AMR gene content, it is vital that the concordance is high between the reference result and the expected outcome (65). The sensitivity is especially important as the misidentification of a resistant strain is more consequential than the finding of silent resistance genes in phenotypically susceptible isolates. As previously mentioned, discrepancies observed between phenotypic reference result and the expected genomic outcome is often due to incorrect phenotypic antimicrobial susceptibility test data.

Assessing the performance of bioinformatics tools is often based on a comparison between the genotypic and phenotypic results and a calculation of the specificity, sensitivity, positive predictive (PPV) and negative predictive values (NPV), accuracy [Simple Matching Coefficient (SMC)] and performance [Matthew's Correlation Coefficient (MCC)] followed by a comparison of these parameter's between the different bioinformatics tools (26, 66).

Surprisingly, only a few studies have benchmarked bioinformatics tools against each other to detect AMR determinants. 24 used two previously published pair-end Miseq datasets (7, 8) of 196 genomes of four species and 143 genomes from two species (five species in total), respectively. Phenotypic susceptibility test data was used as the reference result in predicting AMR determinants when benchmarking the KmerResistance vers 1.0 (target only enzymes) (70% identity and 10% depth corr (co-occurrence of K-mers), ResFinder vers. 2.0 (target only enzymes) [98% identity and 60 coverage (assembly/BLAST)], and SRST2 (90% identity 90% coverage) (clustering/Bowtie2). To further challenge the sensitivity, the datasets were down-sampled to 1% of the reads and re-analyzed. Overall, the three bioinformatics tools performed equally well with almost the same accuracy, SMC and performance, MCC testing the two datasets; SMC and MCC were app. 96% and 0.90 for the Stoesser et al. collection, respectively whereas the SMC and MCC ranged from 98 to 100% and 0.91 to 0.99 for the Zankari et al. collection, respectively with the lowest performance by SRST2 and the highest by KmerResistance (26). The KmerResisance tool performed significant better than the two others when data were contaminated or down-sampled to contain a few reads—all bioinformatics tools performed best using raw reads input data (26).

Another study (ENGAGE) (66) evaluated the Public Health England's GeneFinder tool, which targets enzymes and some chromosomal point mutations for fluoroquinolone resistance using two HiSeq datasets, 125 *Salmonella* genomes and 164 *E. coli* genomes of which a large proportion harbored upregulated *ampC*-mediated resistance to extended spectrum cephalosporins. ResFinder provided the highest accuracy, SMC and performance, MCC predicting resistance in the *E. coli* genomes and GeneFinder for *Salmonella* genomes. The correlation to phenotypic susceptibility testing was for *Salmonella* spp. Ninety percent for all bioinformatics tools but higher for GeneFinder specifically for fluoroquinolones. The accuracy, SMC revealed to be lower in *E. coli* than testing *Salmonella* for all bioinformatics tools due to the bias of the *E. coli* dataset containing a high number of upregulated *ampC* genotypes not predicted by any of the bioinformatics tools (66). Hunt et al. similarly benchmarked the same bioinformatics tools as in Clausen et al. including also the ARIBA tool (30). The ARIBA tool contain in addition to enzymes also chromosomal point mutations thus, outperforming both KmerResistance (26) and SRST2 (37).

Following the benchmarking described above, both the ResFinder and the KmerResistence bioinformatics tools have been updated. Thus, the Resfinder tool now includes a number of chromosomal point mutations such as those to detect resistance to colistin, fluoroquinolones, etc. Overall, the benchmarking exercises revealed that all bioinformatics tools evaluated performed almost similarly good but were affected by the type and quality of input data.

In an assessment of the accuracy of NCBI's AMRFinder, a 2018 study by Feldgarden et al compared it with a 2017 version of ResFinder (33). AMRFinder was evaluated first using a set of 6,242 genomes with 87,679 AST data points for 14 antimicrobial drugs. Overall, 98.4% were consistent with predictions. When compared with ResFinder, most gene calls were identical. While there were 1,229 gene symbol differences, 81% were attributed to differences in database composition. AMRFinder and ResFinder use HMM- and BLAST-based approaches, respectively, and are the commonly used resources for genome-based AMR tracking. Synchronized harmonization of the databases, as is done globally with genomic sequence databases, is needed to minimize inconsistent outputs due to algorithmic differences.

## Ensuring High Quality Genomic Data by Proficiency Testing

Standardization of WGS procedures from DNA preparation to the final genome is paramount to ensure reliable prediction of AMR determinants for surveillance and clinical purposes. To ensure the production of reliable high quality genomic data, laboratories routinely performing WGS should participate in laboratory proficiency testing (PT) or external quality assurance systems (EQAS) (67, 68). For decades, global and regional EQAS in phenotypic AST of foodborne pathogens has been conducted to ensure the quality of performed dilution and diffusion AST (69–71). There is an urgent need to also establish a mechanism to provide a global proficiency testing in the area of WGS to establish standardization in the field (68). This goal is part of the charter of the Global Microbial Identifier (GMI), launched in 2011, to help establish a “global system of DNA genome databases for microbial and infectious disease identification and diagnostics” (https://www.globalmicrobialidentifier.org/).

In 2014, GMI launched its first pilot PT in WGS lead by the DTU and US FDA to trial test the WGS platforms, procedures, test material and the functionality of the assessment pipeline (72). In 2015, a full roll-out of the pilot was delivered by GMI to a global audience. The GMI continued to provide proficiency testing in 2016 and 2017. Cultures and pure DNA for library construction were provided to participating laboratories for DNA purification, library preparation, and WGS followed by *in silico* prediction of wgMLST and AMR determinants. The genomes and analysis were submitted to DTU for quality control assessment using closed genomes of the test strains as a reference. The quality control assessment was facilitated by an in-house developed PT QC pipeline measuring a large number of parameters. These included the numbers of reads after trimming, unmapped reads, map to the total reference DNA, reference chromosome, reference plasmids; proportion of reads that map to reference chromosome; coverage of the reference chromosome and reference plasmids; depth of coverage of total DNA, reference chromosome, and reference plasmids; Phred quality score (Q score), total size and proportion of assembly map to the reference DNA, number of contigs including above a length above 200 bp, N50, and NG50. Underperformance was observed and reported in each trial mainly caused by laboratory contamination or poor performance.

## Data Sharing—Public/Private

**Figure 2:**
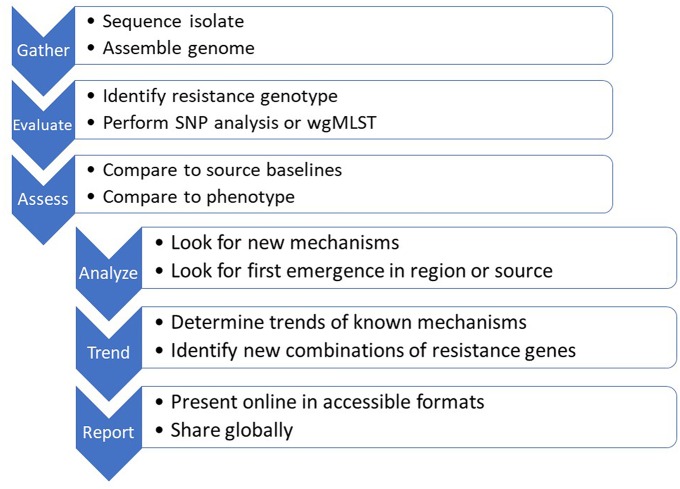
The sequence-based monitoring approach to track global antimicrobial resistance using bioinformatics tools.

An important element of genomics as a tool for AMR surveillance and diagnostics is that, once data quality standards are met, the data set is platform-independent, discrete and portable. The analytical outputs and data sharing then become the most important considerations (Figure 2). A plethora of international and governmental position papers have stressed the need for global cooperation and data sharing to combat infectious diseases and worsening antimicrobial resistance (73–82). Countries have different levels of legal restriction on the sharing of medical information and biological material with potential commercial value or compliance to the EU General Data Protection Regulation. While the legal issue may be more intractable, the public health advantages to global data sharing are obvious. In the US, where fewer restrictions are in place, WGS data from national surveillance systems are continuously placed in the public domain both for public health purposes, and for exploitation by innovators to develop and update new technologies. This permits global access to information on common microbiological threats, something that will become more important as travel and trade increase and as new threats arise.

## Online Repositories to Host and Link Genome and Antimicrobial Susceptibility Data

Concurrently with the vast amount of genomic data being produced, traditional antimicrobial susceptibility testing is being conducted in parallel on a large scale. Up until recently, it was only possible to submit and store DNA sequence data in the International Nucleotide Sequence Database Collaboration (INSDC), whereas all AST data was stored separately in closed local or national repositories. Furthermore, not all genomic data is submitted to the online open genomic repositories of INSDC and shared globally due to difficulties to submit, a lack of appreciation for its value, access to local or national repositories, fear of being data being published by others, or privacy of the data (83). Nonetheless, today the NCBI and the European Bioinformatics Institute (EMBL-EBI) can accommodate AST data along with the WGS information, to facilitate a global monitoring of AMR in bacteria to strengthen global public health (84, 85).

## European Nucleotide Archive Repository

At European Nucleotide Archive (ENA), a mechanism to host and link submitted genomic and AST data has been developed by the EU COMPARE partners and EMBL-EBI (85). Briefly, the EMBL-EBI system allows submitted genomes and associated metadata in the ENA to be stored as open access or privately in a secured login protected repository with named data hubs (86). The system is designed to accommodate submission of susceptibility data from both dilution or diffusion methods. Novel software has been developed to validate conformity of the AST data to ensure harmonization of the data (85). The submitted genomic and AST data could be analyzed by using existing bioinformatics infrastructure and implemented cloud-based bioinformatics workflows in specific an extended version of the Bacterial Analysis Pipeline consisting of ContigAnalyzer-1.0, KmerFinder-2.1, MLST-1.6, ResFinder-2.1, VirulenceFinder-1.2, PlasmidFinder-1.2, pMLST-1.4 (87) with the inclusion of also the cgMLSTFinder 1.0. The submitted data could be queried and downloaded in multiple ways including via the Pathogen Data Portal for surveillance, identification, and investigation https://www.ebi.ac.uk/ena/pathogens/home. Subsequently, the data could be visualized by using a developed Notebook tool integrated the Pathogen Data Portal to query and display all typing data including distribution of the phenotypic AST data enable a potential real time monitoring of AMR (85). The advantage of the data hub model and similar embassy cloud system is the possibility for privacy to control own data having restricted access to only owners or collaborators while analyzing or publishing the data or await less political sensitivity due to GDPR which all a major barriers in data sharing (88–90).

## National Center for Biotechnology Information Repository

The National Center for Biotechnology Information (NCBI) is the US member of the INSDC and part of the United States National Institutes of Health, and houses hundreds of thousands of bacterial genomes from around the world. Sequences are submitted from global research studies, but the majority are from national public health surveillance programs with systematic sampling schema. With the expansion of WGS capacity, the number of genome submission is expected to rise soon to over 100,000 annually from US sources alone.

To help make these large datasets accessible, the NCBI Pathogens page (https://www.ncbi.nlm.nih.gov/pathogens/) was developed. This resource is designed for exploring the genomic features of various bacterial pathogens. These include major foodborne and zoonotic pathogens, such as *Salmonella enterica, Escherichia coli*, and *Campylobacter* spp. Included in these datasets is a variety of metadata, including strain ID, source, date collected, geographical location, antimicrobial resistance, and more. This page was established in collaboration with GenomeTrakr, an international consortium of laboratories organized by the U.S. Food and Drug Administration (FDA) that collect and sequence bacterial strains from a variety of food and environmental sources (91).

A major feature of the Pathogens page is the phylogenetic trees, as genomes are arranged into clusters based on relatedness according to SNPs. These allow users to explore and interpret the relatedness of bacterial strains. These have provided a robust database of bacterial species that can be used for genomic comparisons with isolates collected from human patients. This information can be used to help identify foodborne disease outbreaks and support regulatory actions by the FDA.

Another major aspect of the Pathogens page is the AMR reference gene database mentioned above (33). The tool, AMRFinder is automatically run on all genomes submitted to NCBI, resulting in AMR genotype outputs that identify resistance genes from each sequence (33). This, combined with the phylogenetic tree outputs, allows for identification and potential prioritization of investigations into resistant outbreaks of pathogenic organisms.

The NCBI Pathogens web portal also contains phenotypic information, when submitters of these data choose to include it. Over 7,000 isolates now have phenotypic MIC data associated with them, allowing users to interrogate the data for various resistance phenotypes, including those conferred by mutations not tracked presently by the genotypic outputs of AMRFinder (33).

To help make the resistance information accessible, the US Food and Drug Administration developed a tool called ResistomeTracker (https://www.fda.gov/animal-veterinary/national-antimicrobial-resistance-monitoring-system/global-salmonella-resistome-data). This suite of data dashboards is focused exclusively on analysis and visualization of AMR genes extracted from the complete genomes at the NCBI. ResistomeTracker was developed for the U.S. National Antimicrobial Resistance Monitoring System (NARMS) to better understand the epidemiological aspects of resistance by making the large amounts of resistome data accessible to a broad user audience. This includes the identification of new resistance determinants, differences in the prevalence of resistance genes among various food commodities, and geographical spread over time. Additionally, continuous updates to ResistomeTracker enable users to detect early resistance threats. ResistomeTracker allows for user-directed queries of the data that are informative for individual interests. Because it is linked directly to the NCBI pathogen database, it allows the user to begin a query with a specific resistance allele, and end with a phylogenetic analysis of related strains. It currently is focused on foodborne bacteria, but can be modified to exploit and genome for resistance gene content.

## Using WGS in AMR Surveillance

In the United States, national laboratory capacity for AMR monitoring and WGS is growing. It consists of federally coordinated networks operated by State public health laboratories and Universities. The Centers for Disease Control and Prevention (CDC) coordinates the Antibiotic Resistance Laboratory Network (ARLN) to rapidly detect emerging resistance threats in healthcare, food and the community. Among many activities, this comprehensive network performs WGS for numerous pathogens, including all isolates of *Mycobacterium tuberculosis*. WGS is used also as a routine method to characterize *Neisseria gonorrhoeae*, and other major pathogens, including those involved in outbreaks.

The National Antimicrobial Resistance Monitoring System (NARMS) is a long-standing program focused on bacteria transmitted commonly through food (92). NARMS is a partnership of the CDC, the FDA and United States Department of Agriculture Food Safety and Inspection Service (FSIS); it is focused on tracking resistance in enteric bacteria from humans, retail meats and food animals, respectively. NARMS began systematic WGS of *Salmonella* in 2013 and has incorporated WGS data for *Salmonella* and *Campylobacter* in its reports since 2014. Online tools enable users to examine resistance trends at the genetic level using various query filters. These tools provide graphical visualizations of the genotypes behind changing resistance patterns over time by source and serotype.

As national resistance surveillance matures to better fit the One Health model, animal pathogens and environmental testing are beginning. In the US, the Department of Agriculture National Animal Health Laboratory Network (NAHLN) and the FDA Veterinary Laboratory Investigation and Response Network (Vet-LIRN) are starting to gather resistance information and WGS data on pathogens from food animals and companion animals, respectively. The US Environmental Protection Agency (EPA) conducts periodic water surveys that includes detection of resistance genes. While in the early stages, national public health surveillance programs using DNA sequence information will continue to expand and permit new associations to be inferred from resistomic analyses of the data.

In Europe, its mandatory by law, Directive 2003/99/EC (https://eur-lex.europa.eu/eli/dir/2003/99/oj) for Member States (MSs) to monitor AMR phenotypically by MIC determination in *Salmonella, Campylobacter*, and *E. coli* obtained from healthy food-producing animals and from food. The monitoring also include a specific monitoring of extended-spectrum beta-lactamase (ESBL)-, AmpC- and carbapenemase-producing *Salmonella* and indicator commensal *E. coli* stipulated in the Commission Implementing Decision 2013/652/EU of 12 November 2013 (http://data.europa.eu/eli/dec_impl/2013/652/oj). The data collection on human diseases including AMR from MSs is optimal and based on either MIC or disk diffusion and conducted in accordance with Decision 1082/2013/EU (http://data.europa.eu/eli/dec/2013/1082/oj).

A number of MSs providing data for the specific monitoring of AmpC- and carbapenemase-producing *Salmonella* and indicator commensal *E. coli* from healthy food-producing animals and from food, has expressed an interest to replace the mandatory phenotypic MIC determination with WGS due to this already been implemented locally in the specific MSs. Thus, in the preparatory work of updating the Commission Implementing Decision 2013/652/EU coming into force in 2021, the preliminary draft of the technical specifications on harmonized monitoring of resistance in zoonotic and indicator bacteria from food-producing animals and food from EFSA suggested to allow replacing MIC determination with WGS combined with using the CGE ResFinder tool till 2025 (36). From 2025, the using of WGS combined with using the CGE ResFinder tool will be mandatory for the specific monitoring of AmpC- and carbapenemase-producing *Salmonella* and indicator commensal *E. coli* from healthy food-producing animals and from food and considered to be expended replacing all phenotypic MIC determinations as well as species identification. The resulting AMR determinant profile will be submitted to EFSA and used to predict the phenotype which will be reported in the European Union summary report on antimicrobial resistance in zoonotic and indicator bacteria from humans, animals and food. It will be optional for the individual MSs to also submit the DNA sequences and metadata data to ENA. It's believed that all MSs by 2015 have acquired WGS and conducing bioinformatics analysis of DNA sequences of single isolates for monitoring purposes.

## AMR Surveillance Using Metagenomics

Current AMR surveillance often focuses on few pathogens mainly based on passive reporting of phenotypic laboratory results for a few selected specific pathogens as in the Danish monitoring system, DANMAP https://www.danmap.org/, leading to a narrow pathogen spectrum that does not capture all relevant AMR genes. The majority of AMR genes may be present in the commensal bacterial flora of healthy humans and animals or the environment.

Metagenomics techniques, using short-read next-generation sequencing data, benefit from the ability to quantify thousands of especially transmissible resistance genes in a single sample without any prior selection of which genes to look for. Moreover, it can provide additional information about the presence of bacterial species, pathogens and virulence genes and the data can be re-analyzed, if novel genes of interest are identified.

It was recently shown that metagenomics is superior to conventional methods for AMR surveillance in pig herds (93), useful for comparing AMR across livestock in Europe (94), as well as investigations related to epidemiological data (95). The utility for surveillance of global AMR gene dissemination through international flights (96) and using urban sewage to determine the local and global resistome has also been proven (97, 98).

Metagenomics will sequence all DNA present in the sample including food and host DNA, which may result in low sensitivity. Quantitative PCR procedures, including large scale capture PCR methodologies have been developed, likely providing higher sensitivity (42). However, these methodologies have not been compared with respect to sensitivity and specificity.

In the future the application of metagenomics directly on samples from healthy and clinical ill individuals and animals as well as potential reservoir might results in the ultimate One Health surveillance of AMR allowing determination of all resistance genes and their context in all reservoirs. However, as for single isolates different pipelines and databases are also used for such metagenomics studies and there is a need for global standardization.

## Perspectives

An important advantage of using WGS technologies in detecting and tracking AMR is the opportunity to expand it to align with a One Health surveillance framework and allowing for exact comparisons across reservoirs. This cannot be done using WGS only on the phenotypic antimicrobial class level, but at the exact genetic mechanism level. This One Health goal has so far been impeded by the high cost of testing animal and environmental samples using classical methods based on metabolic and biochemical characterization. As the NGS technology becomes more affordable, it will become more common to use metagenomics to explore the potential role of different environments in the ecology of resistance. Thus, One Health monitoring is now poised to evolve into nucleotide surveillance of complex microbial ecosystems. And to the extent that the data can be generated and reported without delay, it appears that something analogous to a “weather map” of infectious diseases and resistance is possible. This was not practicable in the past, where *ad hoc* gene detection was the norm and PFGE was the typing tool of choice.

## Conclusion

The advancement in whole genome sequencing and the application of online tools for real-time detection of AMR determinants is essential for control and prevention strategies to combat the increasing threat of AMR. We identified a number of accessible tools available in the prediction of AMR determinants to support expanding to establish global pathogen surveillance and AMR tracking based on genomics. In addition, we identified a number of preceding requirements for a successful transition such as curated AMR databases ensuring a high concordance between pheno- and genotypes, benchmarking designs, PT schemes, sharing options etc. There is however, a vital need for standardization of pipelines and databases as well as phenotypic predictions based on the genomic data.

## Author Contributions

RH and PM conceived, outlined and critically revised the manuscript. All authors wrote, read and accepted the manuscript.

### Conflict of Interest Statement

The authors declare that the research was conducted in the absence of any commercial or financial relationships that could be construed as a potential conflict of interest.
